# Evaluation of Safety and Efficacy of Traction, Closed Reduction, and Subsequent Hip Fixation in Hilgenreiner Brace in Patients with Severe Forms of Hip Developmental Dysplasia

**DOI:** 10.1155/2022/8688770

**Published:** 2022-05-29

**Authors:** Ahmad Gharaibeh, Rastislav Sepitka, Jan Pobeha, Daniela Schreierova, Martina Habinakova, Gabriel Vasko, Marek Lacko

**Affiliations:** ^1^Orthopaedic Surgeon, Teaching Department of Orthopaedics and Musculoskeletal Trauma, University of Pavol Jozef Šafárik, Kosice, Slovakia; ^2^Orthopaedic Surgeon, Teaching Department of Orthopaedics and Musculoskeletal Trauma, Univerzitná Nemocnica Louisa Pasteura, Kosice, Slovakia; ^3^Teaching Department of Orthopaedics and Musculoskeletal Trauma, Univerzitná Nemocnica Louisa Pasteura, Kosice, Slovakia; ^4^Medical Information Department, University of Pavol Jozef Šafárik, Kosice, Slovakia; ^5^Teaching Department of Orthopaedics and Musculoskeletal Trauma, University of Pavol Jozef Šafárik, Kosice, Slovakia; ^6^Head of Teaching Department of Orthopaedics and Musculoskeletal Trauma, University of Pavol Jozef Šafárik, Kosice, Slovakia

## Abstract

**Background:**

Hilgenreiner brace (Hb) was developed to improve hip reduction rate and reduce the incidence of femoral head avascular necrosis (AVN). In children under the age of 18 months with unstable hip joints or a dislocated hip joint, the treatment method involves nonsurgical treatment in most cases.

**Objectives:**

To evaluate the effectiveness and safety of traction, closed reduction, and hip fixation in Hb in patients with severe forms of hip developmental dysplasia (DDH) in follow-up.

**Materials and Methods:**

Prospective, clinical, cohort observation and retrospective matched-pair analysis. Analysis of medical records was conducted to evaluate the effectiveness of using Hb for treatment of dislocated hip joints in <18-month-old children. The investigated cases were of the dislocated hip joint since DDH was confirmed through clinical and imaging diagnosis and treated by the application of the close reduction method together with Hb, in a nonhuman position (hip joint in 90 degrees of flexion and 80 degrees of abduction). Analysis was carried out using the modified Berkeley's Mckay criteria and hip joint centralization, and evaluation was done using X-ray images according to the basic modified Severin classification system.

**Results:**

The use of Hb applied after overhead traction to (mean 22.8 days, confidence level (95%)) 68 hip joints showed a significant improvement (92%) in the treated hips. In summary, only one brace replacement was performed due to swelling of the thigh and fixation pressure, three cases suffered from hip joint redislocation after removing the Hb (5%), and one patient had bilateral avascular necrosis (2.8%).

**Conclusions:**

The use of Hb reduced avascular necrosis of the femur head, maintained higher hygiene conditions, and lowered both the risk of cast breakage and skin complications over the use of hip spica as compared to Hb. Hb is more cost-effective, and radiolucency is an additional advantage for this technique. Closed reduction and application of Hb after oral administration of a bolus dose of chlorpromazine chloride or phenobarbital resulted in complication avoidance of total anaesthesia.

## 1. Introduction

**Figure 1:**
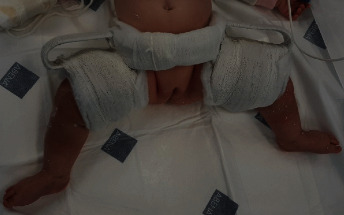
Hilgenreiner brace view from front.

Developmental dysplasia of the hip (DDH) is also known as congenital hip dislocation [1–3]. DDH has a severity spectrum ranging from mild acetabular dysplasia (shallowing) with a stable hip joint to severe forms of hip dysplasia with instability up to dislocation [1–6]. Furthermore, DDH is a concept that represents a continuous developmental process, which varies in presentation and is not always detectable at birth. The treatment method in children under the age of 18 months with unstable hip joints or a dislocated hip joint involves nonsurgical treatment in most cases [1, 5, 7]. Most pathological DDH cases are unilaterally occurring mostly in females and commonly involving the left hip joint. When DDH is diagnosed and treated early, better results are obtained to avoid surgical procedures [1, 3, 5]. The therapy aims to keep the femur head inside the acetabulum, which can be achieved in the flexion and abduction position in the hip joint using Hb (Figure 1) or hip spica [2, 5, 7]. According to Hilgenreiner (1870–1954) [5], the development of these braces is to improve the retention of the dislocated hip joints and to reduce the incidence of avascular necrosis of the femoral head (AVN) [5].

In all DDH cases, the acetabulum is shallow, where the femur head cannot be firmly held and folded into the acetabulum. In some instances, the ligaments maintaining the correct hip position are lax [1, 3, 8].

The degree of hip joint instability or instability varies in children with DDH. Dislocation in the most severe cases of DDH includes the femoral head being completely dislocated from the acetabulum. USG according to Graf types III and IV. Dislocatable tendency means the femoral head lies inside the acetabulum but may easily dislocate during a clinical examination. Subluxation means the femoral head is not completely dislocated but can easily be moved to the acetabulum during a clinical examination [1, 3, 4, 9–12].

The clinical examination of children is based on McKay's criteria modified by Berkeley [13]. The criteria indicate that hip mobility in the sagittal plane (flexion/extension) is 130-0-10°, in the frontal plane (abduction/adduction) is 40-0-30 degree, and in the transverse plane (extrarotation/intrarotation) is 35-0-35 degree. The criteria also state hip joint palpation, Trendelenburg test, neurovascular examination, limb length comparison, hip stability, and limping [1, 3, 5, 12–16].

### 1.1. Objectives

Hb was developed to improve the hip reduction rate and reduce the incidence of femoral head avascular necrosis (AVN). This study aims to evaluate the effectiveness and safety of the Hb.

## 2. Methods

The study design was prospective, clinical, cohort observation and retrospective matched-pair analysis. The place of study was Louis Pasteur University Hospital in Košice with 1356 beds. This hospital is the second largest hospital that provides high-quality health care in specific fields for Eastern Slovakia. The study population was as follows: all neonates with displaced or unstable hip joints at the USG Orthopaedics and Traumatology Clinic were examined according to IId, III, and IV types using Graf classification. The total number of patients followed up was 54 children (71 hip joints) with severe hip dysplasia (stages IIc–IV), of which 11 were males and 43 were females. Exclusion criteria were as follows: children with secondary hip dysplasia, based on neurological, myopathic, or other connective diseases, were excluded from this study due to the therapeutic policy and timing [1, 3]. Inclusion criteria were as follows: DDH diagnosis was performed as a standard by a clinical examination, ultrasound examination, and X-ray in the anteroposterior and axial view (Lowenstein Frog view) in 0–18-month-old children. The patient's parents were instructed on the Hb treatment protocol, and informed consent was obtained from all parents or legal guardians. All cases were recorded from 2003 to 2014. The use of the Hb is an effective and safe method for hip retention. Artery circumflexion of the femur is under pressure on the pineal gland and hip joint (checked). Hb is more advantageous over gypsum spica for patient hygiene. Data were collected from hospital archives. As part of the study, patients were invited for a follow-up clinical examination and compared to a control group from a Chinese study conducted in multiple clinical centers. The control group sample consisted of 39 patients (51 hip joints) treated with closed reposition, followed by hip replacement in a human position, and checked after 24 to 36 months for redislocation and avascular necrosis of the femoral head YiQiang et al. [10]. The Chinese age group was younger than the Slovak age group, where both groups were of walking age. A retrospective study was carried out to evaluate medical records of all children under 18 months using USG and X-ray diagnosed with dislocated hip joint [8, 17–19] due to DDH and treated with closed reduction and Hb application by 3 paediatric orthopaedic surgeons with a mean age of 31.5 weeks. All admitted patients were treated using vertical traction for approximately 3 to 5 weeks according to the clinic's internal protocol (0.25 kg weight for both lower limbs was applied on the first day and then we added 0.75 kg). Before operation, all patients orally received a bolus dose of chlorpromazine chloride or phenobarbital. Subsequently, in the operating room under X-ray (C arm), a closed reduction and Hb application, wrapped in a narrow plaster in the thigh area without affecting the gluteal area, were performed, without general anaesthesia. The brace was applied at 90 degrees of flexion and 80 degrees of abduction in the hip joint under the C arm control. On the following day, a control X-ray was taken and the children (usually accompanied by his mother) were released into outpatient care. Outpatient check-up was carried out every two weeks during a three-month treatment period using the Hb. The Hb was removed on an outpatient basis after 12 weeks using an electric saw or brace cutter, followed by loading of the abduction brace for an additional twelve weeks.

The basic modified Severin classification system was applied to assess the centralization of the hip joint using the Hilgenreiner line, the Perkin line, and the Shenton line on X-rays [11, 13, 17]. In case of hip dislocation, the femur head has delayed ossification.

The acetabular index (the angle formed by the line drawn from the point on the medial triradiate cartilage to the point on the lateral edge of the acetabulum and the Hilgenreiner line) was maintained at <25° in patients older than 6 months. The centre edge angle (CEA-Wiberg) is the angle formed by a vertical line drawn through the centre of the femur head and a line drawn from the centre of the femur head to the lateral edge of the acetabulum and was maintained at<20°. Measurements and assessments were considered reliable only in patients older than 5 years. Finally, other changes such as lateralisation or widening of the medial joint space were observed. Irregular ossification of the femoral head and reduced size of the ossifying nucleus were considered as subchondral fractures [1, 4, 15, 17].

Treatment complications (redislocation, swelling, and neurovascular parameters) and their related risks according to the child's age were evaluated.

### 2.1. Statistical Analysis

SPSS software and Excel were used for statistical analysis and processing of the collected data. Descriptive statistics of the data including frequencies (N), arithmetic mean (AM), standard deviation (S), standard error of estimation (SE), median (Mdn), mode, sample variance (S^2^), coefficients of skewness and sharpness, minimum and maximum values, and range of values were used. Prior to statistical tests, the normality of the distribution of individual dependent variables was tested based on the coefficients of obliquity and sharpness, respectively, and their Z-score (Field, 2013). Since the assumption of normality was not confirmed, a nonparametric test, namely, the Mann–Whitney *U* test, was used to find the statistical significance of the relationship between a categorical and an ordinal variable. In this case, it was a question of verifying the relationship between a categorical variable and a continuous variable with a nonnormal distribution.

## 3. Results

The total number of patients treated with the closed reduction method and Hb fixation was 54 children (71 hip joints) with developmental severe hip dysplasia (stages IIc–IV using Graf classification), of which 11 were males and 43 females (79.6%). The Chi-square test showed that the difference in gender distribution between the Chinese group and the study group was not significant: *χ*^2^ = 2.3126, *p*=0.128. The follow-up examination performed by the main author in 2018-2019 (children of 4–16 years of age) showed that all patients were subjective without difficulty and without a history of pain or limping according to the parents. Based on clinical examination according to McKay's criteria modified by Berkeley [18] in Table 1, all patients belonged to the first-degree (excellent) full-range hip mobility: in the sagittal plane (flexion/extension), 130-0-10 degrees, in the frontal area (abduction/adduction), 40-0-30 degrees, and in the transverse plane (extrarotation/intrarotation), 35-0-35 degrees. The basic Severin classification system was used to evaluate X-rays of patients in AP projections of the cox (Table 2) [1, 13, 17, 20].

The hip joints were palpably painless, the Trendelenburg test was negative, acrals were without neurovascular deficit, there was no recording of limb length discrepancy, and there was a stable hip joint without limping [3, 9, 12, 14, 21]. For hip joints with severe dysplasia (71 hip joints), 17 cases were bilateral (31.5%), with only the right side (26) (48.1%) and the left side (20.4%). The Side-Chi-square test showed that the difference in the distribution of pages is not significant between the Chinese group and the study group: *χ*^2^ = 2.0483, *p*=0.359.

During the follow-up in 2018-2019 (the average follow-up period is 10 years (4–16 years)), an improved finding based on clinical examination and X-ray of 44 treated patients (59 hip joints) was observed. The Hb was applied after overhead traction in 68 hip joints, and excellent results were obtained in 62 hips (92.2%). Two children (3 hips) showed up for a follow-up after the application of Hb; in one patient, Hb was removed due to swelling of the thigh and fixation pressure. In three patients, the hip joint redislocated after brace removal, but the condition improved after reoperation and prolongation of the treatment period using the Hb. In the control group, there was a redistribution of the hip joint after removing the spike of 12 hip joints out of 51 (23.5%). One 13-year-old patient developed bilateral coxa plana (avascular necrosis). However, 4 years after the Hb treatment with left side DDH, only one patient showed no clinical symptoms, an active football player, and his mother refused to deal with his stable condition even though he had one side of DDH.

In the Chinese control group, there were 4 (7.8%) patients who developed avascular necrosis in 4 hip joints. According to the basic Severin classification system, one patient belonged to the second class and the other patients belonged to the 1^st^ grade (Table 2). Three patients were not possible to be contacted. McKay's criteria included all children in the first grade, and by using the Severin classification, only data from final outcomes were compared.

The Chi-square test showed significant differences between the control (Chinese) and Slovak groups: *χ*^2^ = 22.6866, *p* < 0.001.

A statistically significant difference was observed between patients with and without complications in the number of days that elapsed from birth to admission to the hospital: *U* = 56,000, *p*=0.026. Patients with complications have a significantly higher number of days from birth to hospital admission (age of children: 4.11 ± 2.04 and range: 1–10 months) than patients without complications.

**Figure 2:**
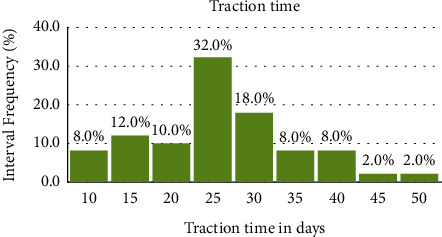
Vertical traction time before application of the Hilgenreiner brace.

**Figure 3:**
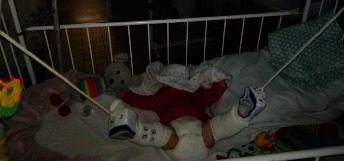
Overhead traction.

It is clearly evident that that there is a statistically significant relationship between the age of children and the occurrence of complications. The redislocation Chi-square test showed significant differences in the closed question type (yes and no answers) between the Chinese group and Slovak group: *χ*^2^ = 2.7067, *p*=0.09. The time of vertical traction according to the internal adopted protocol and to the clinical examination of hips of children was 3–5 weeks (up to 4 weeks for about 80% of patients). Extremely long duration of vertical traction was in 12% of patients due to their general health and clinical hip condition at trial reduction under X-ray (C arm) (Figures 2 and 3).

A statistically significant relationship was observed between the number of days from birth to hospital admission and the onset of complications using the Mann–Whitney *U* test. The relationship between the categorical variable “occurrence of complications” and the continuous variable with nonnormal distribution of data “number of days from birth to hospital admission” was analyzed by examining the differences in days from birth to hospital admission between groups of patients with and without complications (Table 3).

Compared to the YiQiang study, all tests were performed at a significance level of 0.05. A *t*-test for independent groups and a nonparametric Chi-square test were applied. Comparison of age using a *t*-test for independent groups showed that there is a significant difference between the Chinese group and Slovak group: *t* (90) = 42.3084, *p* < 0.001.

## 4. Discussion

DDH is one of the most serious and important congenital abnormalities of the musculoskeletal system, as it accounts for 6.9% of all congenital abnormalities [1–3, 12, 18]. Due to screening, DDH is often detected almost in newborns [2, 11, 12, 15]. Although Hb has been used for the past 9 decades in almost all departments of paediatric orthopaedics in Czech and Slovakia [5], it has not been studied previously.

Closed reduction and application of the Hb are used in the case of Frejka or Pavlik harness devices which were not effective due to severe DDH grades. Developmental dysplasia of the hip (DDH) with dislocation affects 0.1% of neonates; however, the overall incidence of DDH is 0.48% [6, 11, 12].

In Central Europe, 3-4% of newborns had DDH although with dislocation affecting 0.2% of newborns [6, 21]. Compared with Bitar's study (2014-2015), which retrospectively evaluated the use of hip spica in 21 children, 42 patients had hip spica and one patient had a gluteal pressure ulcer [22]. In this study, no complications were observed in our group (due to the nonhuman position of the hips in Hb) (Slovak group). Another study using a DiFazio hip spica in 297 children in the United States (77 children), representing 28% of patients, had a skin complication, indicating repeated anaesthesia and additional costs for plastering and hospitalization of patients [7, 9, 10, 14, 15, 19, 22]. In this study, only one patient had swelling of the thigh after the Hb application. Plastering without anaesthesia in the operating room reduced the cost of hospitalization.

Waggan (2009) reported similar results in Pakistan [23], where 25% of patients with DDH were males (21% in this study). Waggan (2009) observed 25% of hip joint redislocations after hip spica application, while in this study, only 5% of the patients suffered redislocation.

Similar results were also reported in China after the use of a hip spica in 39 children (51 hip joints) YiQiang et al. [10].

Due to exposure of the gluteal area and the free movement of the knees and ankles, the Hb is also more advantageous than a hip spica from a child hygiene perspective. In addition, the lighter weight of Hb which enables easier baby carrying, absence of skin injury, and plaster breakage makes the Hb a preferable method for DDH treatment and recovery.

Furthermore, the risk of avascular necrosis (AVN) after Hb treatment was reduced from 35% to 13% with a hip spica and from 5% [10] to 2.8% in the YiQiang et al. control group.

The time of vertical traction and subsequent application of the Hb reduces the incidence of avascular necrosis of the femoral head, which agrees with our group (Slovak group) [18, 19]. Worldwide, approximately 80% of females were affected by DDH. The time of vertical traction and subsequent application of the Hb reduces the incidence of avascular necrosis of the femoral head, which agrees with the Slovak group. Although the extremely long duration of vertical traction was in 12% of the patients in the Slovak group due to their exaggerated general health condition, we can judge that the extremely long duration of overhead traction is safe based on the obtained results (2.8%). The follow-up examination and X-ray were evaluated by the main author only without MRI assessment like the recommendations in the Chinese study (Yong et al., 2018) [7, 8].

### 4.1. Limitation

Study limitations exist and can influence the interpretation of the findings. The records are obtained from different centres and different surgeons (one centre in both China and Slovakia in addition to 3 paediatric orthopaedic surgeons). Other imaging methods such as arthrography, CT, and MRI were not performed on the study cases (patients). The authors believe that imaging methods are considered as additional examinations depending on the culture of the workplace in the teaching department of Louis Pasteur University Hospital.

## 5. Conclusion

Conservative treatment of severe developmental hip dysplasia after traction using closed reduction and loading of Hb is most effective in patients younger than 18 months. The incidence of avascular necrosis of the femoral head is very rare (2.8% of all cases). The child's hygiene is significantly better than with a plaster spike. The weight of the brace is much lower, and therefore, carrying the baby is easier. Skin injury and plaster breakage are lower with the Hb. Closed reduction and loading of the Hb after administration of a bolus dose of chlorpromazinium chloride or phenobarbital orally does not require intubation of the patient, and complications of general anaesthesia can be avoided. The time of vertical traction and subsequent loading of Hb reduces the incidence of avascular necrosis of the femur head. According to the obtained results, it is advised to use Hb compared to hip spica. The current treatment method by prolonged traction time in the hospital (1-2 weeks) before Hb application in severe DDH grade treatment increases hospitalization costs, but it is very effective and reduces complications.

## Figures and Tables

**Table 1:** McKay's criteria modified by Berkeley.

Degree	Grade	Description
I	Excellent	Stable, no pain, no limping, Trendelenburg test was negative, and complete hip movements
II	Good	Stable, no pain, limping, Trendelenburg test was negative, and mild deficient of hip movements
III	Fair	Stable, no pain, limping, Trendelenburg test was positive, and deficient of hip movements
IV	Weak	Unstable, painful, limping, and Trendelenburg test was positive

**Table 2:** Basic Severin classification system.

Class	X-ray appearance	Angle of the central margin
I	Normal	15°–25°
II	Medium deformity of the femoral head, neck of the femur, or the acetabulum	15°–25°
III	Dysplasia without subluxation	<15° (6 to 13 years) <20° (>14 years)
IV	Subluxation	>0°
V	The femoral head is articulated with a pseudoacetabulum in the proximal part of the original acetabulum	
VI	Redislocation	

**Table 3:** Differences between patients with and without complications in the number of days that elapsed from their birth to hospital admission.

	Number of days from birth to hospital admission					
	Discrepancy			Mann–Whitney *U* test		
Occurrence of complications	*N*	AM	SD	*U*	*Z*	*p*
No	43	112,98	56,294	56,000	−2.227	0.026
Yes	6	380,50	516,397			

AM: arithmetic mean; SD: standard deviation; *U*: Mann–Whitney *U* test; *Z*: standard score for Mann–Whitney *U* test; *p*: level of statistical significance.

## Data Availability

The Excel sheet data used to support the findings of this study are available from the corresponding author upon request (gharaibeh@seznam.cz). All data generated or analyzed during this study are included in this published article.
